# Riedel's Thyroiditis: Report of Two Cases and Literature Review

**DOI:** 10.1155/2019/5130106

**Published:** 2019-12-09

**Authors:** Victor Manuel Blanco, Camilo Andrés Páez, Angela María Victoria, Luis Guillermo Arango, Ana María Arrunategui, Juliana Escobar, Veline Martínez, Guillermo Edinson Guzmán

**Affiliations:** ^1^Internal Medicine Unit, Fundación Valle del Lili, Cra 98 #18-49, Cali 760032, Colombia; ^2^Health Sciences Faculty, Universidad Icesi, Fundación Valle del Lili, Cra 98 #18-49, Cali 760032, Colombia; ^3^Internal Medicine Unit – Department of Endocrinology, Fundación Valle del Lili, Cra 98 #18-49, Cali 760032, Colombia; ^4^Pathology Surgery Unit, Fundación Valle del Lili, Cra 98 #18-49, Cali 760032, Colombia

## Abstract

Riedel's thyroiditis is a rare entity consisting of a fibrotic process of the thyroid which can generate gland destruction, infiltration of cervical structures and even airway obstruction. It has been associated with systemic fibrotic disorders, autoimmune diseases, and more recently with spectrum of diseases related to excess of Immunoglobulin G type 4 (IgG4). Two cases of Riedel's thyroiditis by IgG4, confirmed by immunohistochemistry and was managed surgically with favorable results during the follow-up time, are presented. These case descriptions highlight the diagnostic challenge of this disease, describe the response with surgical management, and make a brief update on the subject.

## 1. Introduction

Riedel's thyroiditis is a fibrotic process of the thyroid gland which characteristically extends through adjacent tissues generating gland destruction, infiltration in cervical structures and even air way obstruction. It is a rare condition, with a prevalence of one per 100.000 inhabitants, which affects more frequently women between 30 and 50 years of age. The precise etiology of this disorder is not still clear. It has been described an association with systemic fibrotic processes, autoimmune diseases, and more recently with diseases from the spectrum of excessive immunoglobulin G type 4 (IgG4).

Regarding the treatment for this condition there is no international consensus, but it has been described the surgical approach, use of systemic steroids and inmunosupressants like tamoxifen, rituximab, and mycophenolate mofetil, among others with varying rates of success. We describe two cases, in which diagnosis was confirmed by immunohistochemistry and additionally managed with surgical intervention, with satisfactory results until the follow-up date.

## 2. Case Presentation

### 2.1. Case 1

**Figure 1:**
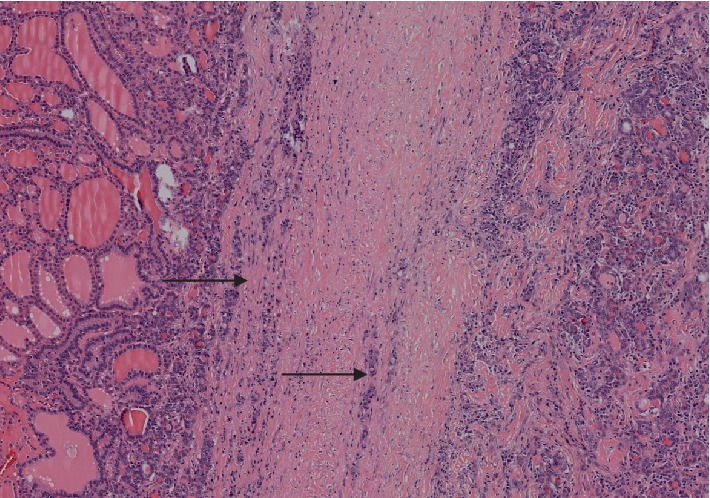
Thyroid gland pathology. Hematoxylin and eosin stain of histological sample from case 1, showing fibrosis and tissue distortion. Original magnification ×20.

**Figure 2:**
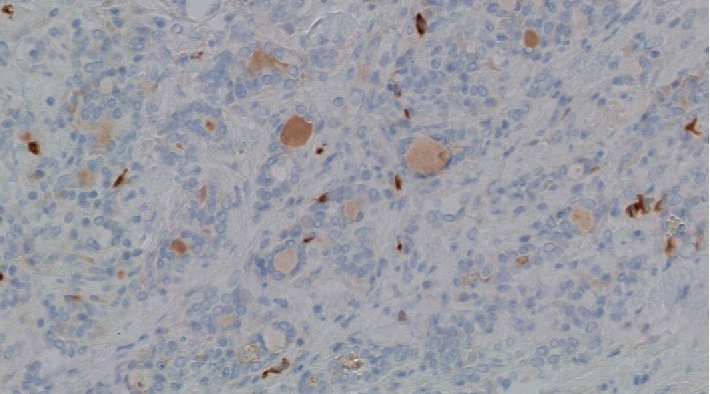
Thyroid gland pathology. Staining for IgG4. Multiple IgG4-positive cells were observed (brown color). Original magnification ×40.

A 38-year-old female from Palmira, Colombia, without past medical history presented with a nonpainful mass in the anterior neck region of nine months of evolution associated to dysphagia, hoarseness, and dyspnea of night predominance. Physical examination revealed good general condition, no areas of swelling or local inflammation, and vital signs in normal ranges. A grade III goiter was palpable mainly in the right thyroid lobe, its diameter was about five centimeters and had a hard consistency without pulsatility or murmurs. Thyroid-stimulating hormone (TSH) test was reported as 0.153 *μ*IU/mL (normal range 0.4–4.5), free T4 1.19 ng/dL (normal range 0.8–1.7), and negative antithyroid peroxidase (anti-TPO) antibodies. In addition, serological immunoglobulins were tested resulting in IgG 905 mg/dL (normal range 767–1590), IgM 150 mg/dL (normal range 37–286), and IgA 200 mg/dL (normal range 61–356). The patient had no signs or symptoms of hyperthyroidism; therefore, this condition was interpreted as subclinical hyperthyroidism. Fine-needle aspiration biopsy (FNAB) was taken from the right thyroid nodule with unsatisfactory results (Bethesda I). The patient was taken to total thyroidectomy; surgical findings depicted a five centimeters complex mass with hard consistency, whitish appearance and a thick capsule that compressed and deformed the trachea. Its histological study identified thyroid gland with follicles of different sizes, without oxyphilic changes, full of colloid material surrounded by abundant dense fibro-connective tissue with interspersed collagen and abundant lymphoplasmacytic inflammatory infiltrate with some eosinophils (Figure 1). The fibrous area constituted the majority of the nodular lesion, without evidence of malignancy, findings that corresponded to Riedel's thyroiditis. Immunohistochemistry tests were performed, which were positive for IgG4 (Figure 2). A favorable postsurgical evolution was obtained, achieving an euthyroid state with levothyroxine substitution.

### 2.2. Case 2

**Figure 3:**
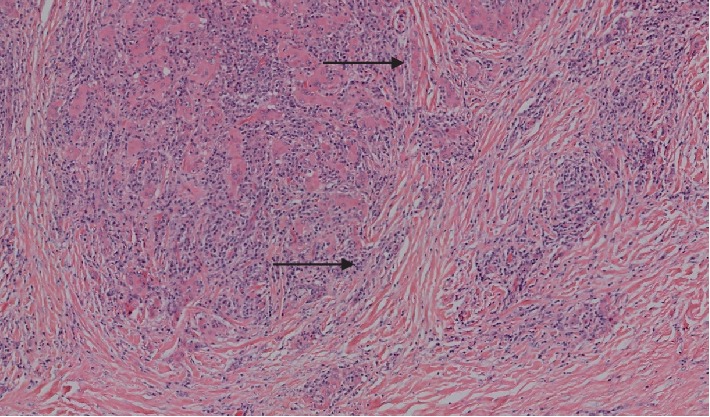
Thyroid gland pathology. Hematoxylin and eosin stain of histological sample from case 2, showing fibrosis and tissue distortion. Original magnification ×20.

A 56-year-old male patient with medical history of hypertension, chronic obstructive pulmonary disease, and chronic kidney disease on renal replacement therapy, presented with a right cervical mass with progressive growth associated to dyspnea and dysphagia to solid foods. At physical examination the patient had vital signs in normal ranges; also, presented a large thyroid mass of right predominance with continuity up to the thoracic operculum, of approximately 12 cm in diameter and hard consistency without associated adenopathies. Initial TSH value was 269 *μ*IU/mL and substitution with levothyroxine was started. Thyroid ultrasound showed enlarged thyroid lobes, with multiple nodular hypoechoic and hyperechoic images with irregular edges up to 2.5 cm. Through fibrobronchoscopy, extrinsic compression of the trachea was documented at three centimeters from the glottis, with occlusion of 20% of the luminal space, for which thyroidectomy was decided. During surgery, a large thyroid gland was visualized with an inflammatory, fibrotic, and multinodular aspect. The pathology report described a gland of 177.5 grams, of a dark-brown color, and a smooth surface, to which cuts were made, finding that the entire gland had fibrotic and multinodular aspect. Microscopic visualization showed tissue with architectural distortion due to the presence of extensive fibrosis, with severe atrophy of the follicles, evident dense inflammatory infiltrate and abundant plasma cells (Figure 3). Immunohistochemical study helped to confirm the diagnosis of Riedel's thyroiditis, with more than 10 IgG4-positive plasma cells per high-power field (HPF), with an IgG4/IgG ratio greater than 40%.

## 3. Discussion

Riedel's thyroiditis is a fibrotic process associated with infiltration by mononuclear inflammatory cells that compromise thyroid gland but usually extends beyond it invading the adjacent soft tissue. Fibrous involvement can extend to the recurrent laryngeal nerves causing dysphonia, towards the trachea causing compression or stenosis, towards the parathyroids causing hypoparathyroidism, or other tissues such as the mediastinum and chest wall. This invasive capacity differentiates Riedel's thyroiditis from other forms of infiltration of the thyroid gland. Epidemiologically, it is a rare disorder that affects more women than men, with an incidence of approximately one case per 100.000 patients, most frequently seen between the fourth and sixth decades of life [1].

To date, the etiology of Riedel's thyroiditis remains unknown, in some cases it has been considered as the thyroid manifestation of multifocal fibrosclerosis, which is partly supported by its association with other fibrotic disorders (retroperitoneal fibrosis, fibrosing mediastinitis, sclerosing cholangitis, among others); however, evidence for this association is scarce [2, 3]. The autoimmune etiology has also been suggested due to the histopathological findings of mononuclear infiltrate, vasculitis, and high prevalence of anti-thyroid antibodies in affected patients, in addition to the association with disorders such as Hashimoto's thyroiditis, Addison's disease, type I diabetes, Graves' disease, and pernicious anemia [4].

It is known that Riedel's thyroiditis can occur as part of systemic diseases associated with IgG4, being characteristic in these cases the tissue infiltration by IgG4 positive plasma cells, as well as elevated serum levels of this immunoglobulin, although normal serum levels can be seen in up to 30% of patients [5–7]. In none of our cases the presence of extra thyroid fibrosis was documented.

From the clinical point of view, there are no pathognomonic findings in Riedel's thyroiditis [8, 9]. Regarding the clinical manifestations of this disorder, goiter is usually found, which can vary in size but typically has a stony consistency and equally affects both thyroid lobes, it is usually slow-growing and in most cases is asymptomatic, although some patients may report dyspnea, dysphagia, dysphonia, or symptoms and signs of superior vena cava syndrome or lower respiratory infection (obstructive pneumonia secondary to compression created by the growing mass) [10]. The adjacent involvement can extend to cervical lymph nodes and can be accompanied by adenopathies attached to deep planes that may suggest a diagnosis of neoplastic etiology. In terms of symptomatic presentation, Fatourechi et al. found pain (24%), dysphagia (33%), vocal cord paralysis (29%), and tracheal narrowing (48%) [11]. In our experience, the cardinal symptoms were mainly respiratory due to extrinsic compromise of the airway.

Regarding diagnostic imaging strategies, scintigraphy usually shows a heterogeneous uptake, while computer tomography (CT) and nuclear magnetic resonance (NMR) show the extensive fibrosis of the thyroid parenchyma. The advantage of CT and NMR is that they can assess the extent of compromise to other structures of the neck, including the possibility of vascular invasion. Thyroid and neck ultrasound can also be helpful, showing multiple hypoechoic focal lesions, which lack blood flow when evaluated by color doppler mode [12]. Other imaging modalities that have been described include positron emission tomography with the use of fluorodeoxyglucose (PET-Scan), which shows areas of increased metabolism, modality that can be used for monitoring the disease activity [13].

Most patients have normal thyroid function, both TSH and Free T4 are normal in the vast majority of those affected, although it is reported that up to 30% have hypothyroidism [8]. In a 30-year retrospective analysis, Fatourechi et al. reported at the Mayo Clinic 17 hypothyroid patients and only one with hyperthyroidism out of the 22 total studied patients [11]. On the other hand, it is useful to perform the measurement of thyroid autoantibodies due to more than 50% of patients have high levels [8, 9]. In our case, the first patient had subclinical hyperthyroidism and negative anti-TPO antibodies and the second one exhibited hypothyroidism and did not have a report of antithyroid antibodies.

As to the histopathological findings, the sample can be obtained by both open surgery plus biopsy or FNAB, being the latter less helpful in Riedel's thyroiditis compared to other pathologies of the gland, since in many patients the sample is inconclusive evidencing only fibrosis and mononuclear infiltrate with few cells of thyroid epithelium, which is difficult to differentiate from other disorders with similar presentation like subacute thyroiditis, the fibrous variant of Hashimoto thyroiditis (where non-producing IgA plasma cells predominate), or the paucicellular variant of anaplastic thyroid carcinoma [14]. It is precisely due to the foregoing that many of the diagnostic confirmations require surgical removal, in which hard, whitish, and avascular tissue can be evidenced macroscopically.

In advanced stages, the predominant findings under the microscope are tissue fibrosis, absence of thyroid follicles and poor lymphocyte-predominant cellularity, being also characteristic the perithyroid infiltration of adipose tissue, vessels (even with associated thrombosis), nerves, and even the trachea and muscles. In up to one fourth of the patients, it can also be seen hemorrhagic cysts within the affected parenchyma. Deep tissues can be so compromised that in several cases complete removal of the gland becomes impossible. Thus, the trained eye of the pathologist will search for clues that suggest the diagnosis while, at the same time, could discard other probable etiologies such as malignancy or granulomatous entities, being of special interest the differential diagnosis with anaplastic paucicellular thyroid carcinoma, that may have a similar pattern of fibrosis and invasion to adjacent planes, in addition to characteristic tapered cells that can be detected by using immunohistochemistry for specific makers of the thyroid line [15].

Due to the low incidence of Riedel's thyroiditis, there are no guidelines or large clinical studies that refer to the optimal management of the condition, in addition, although most cases are progressive, there are reports of patients in whom the disease stabilizes or even reverses. Most authors agree that the therapy should be aimed at treating hypothyroidism in those who exhibit it and at managing the fibrosclerotic manifestations that can put the patient's life at risk (e.g., compression of the airway that may lead to recurrent pneumonias).

There are multiple approaches for the treatment of fibrosis, being systemic steroids, the first line of treatment given to the majority of patients, which decreases the size and hardness of the goiter in only a few of the patients, usually, being necessary prolonged therapy since it is not uncommon to lose the improvement achieved once the doses are titrated downwards [16]. Tamoxifen is another agent used with successful reports, at doses of 10–20 mg every 12 hours. There are descriptions with significant decrease in goiter size in patients who previously did not respond to steroids; the exact mechanism by which this improvement is achieved is not known in detail, but it would seem to be associated with reduction in fibroblast proliferation mediated by transforming growth factor beta (TGF-B) [17].

Regarding surgical management, the indications are basically two: sampling for histopathological diagnostic confirmation, as previously described, and compression or stenosis relief at the level of trachea or esophagus. It has to be considered that the management in these cases should be limited to the resection of the segment that is generating obstruction. The complete resection of the gland is inadvisable due to the high possibility of damaging nearby structures such as the parathyroid and recurrent laryngeal nerves because of the lack of margins of resection. In totally refractory cases, low-dose radiation therapy has been used as a final management, but there is not concrete information about its efficacy [18, 19].

## 4. Conclusion

As it has been depicted in this review, Riedel's thyroiditis is a rare entity, scarcely described, with characteristics that suggest association with systemic fibrosis and other disorders triggered by IgG4. Diagnosis can be challenging due to the unknown etiology, and the unspecific symptoms that overlap with other disorders. As to the treatment, there are no guidelines or general consensus, and the proposed treatments do not go beyond case reports or small case series. The aim of our publication was to describe the cases that occurred in the institution Fundación Valle del Lili in Cali, Colombia; diagnosed by histopathology and managed with surgical intervention, where good clinical evolution was achieved.
